# Prediction of daily mean and one-hour maximum PM_2.5_ concentrations and applications in Central Mexico using satellite-based machine-learning models

**DOI:** 10.1038/s41370-022-00471-4

**Published:** 2022-09-10

**Authors:** Iván Gutiérrez-Avila, Kodi B. Arfer, Daniel Carrión, Johnathan Rush, Itai Kloog, Aaron R. Naeger, Michel Grutter, Víctor Hugo Páramo-Figueroa, Horacio Riojas-Rodríguez, Allan C. Just

**Affiliations:** 1 Department of Environmental Medicine and Public Health, Icahn School of Medicine at Mount Sinai, New York, NY USA; 2 Department of Environmental Health Sciences, Yale University School of Public Health, New Haven, CT USA; 3 Center on Climate Change and Health, Yale University School of Public Health, New Haven, CT USA; 4 Department of Geography and Environmental Development, Ben-Gurion University of the Negev, Beer Sheva, Israel; 5 Earth System Science Center, University of Alabama in Huntsville, Huntsville, AL USA; 6 Instituto de Ciencias de la Atmósfera y Cambio Climático, Universidad Nacional Autónoma de México, Ciudad de México, México; 7 Comisión Ambiental de la Megalópolis, Ciudad de México, México; 8 Dirección de Salud Ambiental, Instituto Nacional de Salud Pública, Cuernavaca Morelos, México; 9 Institute for Exposomic Research, Icahn School of Medicine at Mount Sinai, New York, NY USA

**Keywords:** Machine-learning model, Environmental modeling, Particulate matter, Remote sensing, Air quality management, Air pollution

## Abstract

**Background:**

Machine-learning algorithms are becoming popular techniques to predict ambient air PM_2.5_ concentrations at high spatial resolutions (1 × 1 km) using satellite-based aerosol optical depth (AOD). Most machine-learning models have aimed to predict 24 h-averaged PM_2.5_ concentrations (mean PM_2.5_) in high-income regions. Over Mexico, none have been developed to predict subdaily peak levels, such as the maximum daily 1-h concentration (max PM_2.5_).

**Objective:**

Our goal was to develop a machine-learning model to predict mean PM_2.5_ and max PM_2.5_ concentrations in the Mexico City Metropolitan Area from 2004 through 2019.

**Methods:**

We present a new modeling approach based on extreme gradient boosting (XGBoost) and inverse-distance weighting that uses AOD, meteorology, and land-use variables. We also investigated applications of our mean PM_2.5_ predictions that can aid local authorities in air-quality management and public-health surveillance, such as the co-occurrence of high PM_2.5_ and heat, compliance with local air-quality standards, and the relationship of PM_2.5_ exposure with social marginalization.

**Results:**

Our models for mean and max PM_2.5_ exhibited good performance, with overall cross-validated mean absolute errors (MAE) of 3.68 and 9.20 μg/m^3^, respectively, compared to mean absolute deviations from the median (MAD) of 8.55 and 15.64 μg/m^3^. In 2010, everybody in the study region was exposed to unhealthy levels of PM_2.5_. Hotter days had greater PM_2.5_ concentrations. Finally, we found similar exposure to PM_2.5_ across levels of social marginalization.

**Significance:**

Machine learning algorithms can be used to predict highly spatiotemporally resolved PM_2.5_ concentrations even in regions with sparse monitoring.

**Impact:**

Our PM_2.5_ predictions can aid local authorities in air-quality management and public-health surveillance, and they can advance epidemiological research in Central Mexico with state-of-the-art exposure assessment methods.

## Introduction

Fine particulate matter with aerodynamic diameter ≤2.5 microns (PM_2.5_) affects more people than any other pollutant, and has been consistently associated with mortality and morbidity from cardiovascular and respiratory causes [1]. Over the last decade, epidemiological evidence has related PM_2.5_ to many other health outcomes, such as cardio-metabolic diseases (including diabetes, hypertension, metabolic syndrome), neurological disorders (stroke, dementia, Alzheimer’s disease, autism, Parkinson’s disease), and perinatal outcomes (premature birth and low birth weight) [2]. At the same time, exposure scientists have developed new modeling approaches for air-pollution epidemiology, moving away from the use of data from ground monitors alone. Interest has grown in models using remote-sensing products, particularly aerosol optical depth (AOD) for the prediction of ground level PM_2.5_ concentrations at high spatial resolutions, such as 1 × 1 km. AOD is a measure of the amount of light absorbed and scattered throughout the atmospheric vertical column by the collection of suspended particles (e.g., urban haze, smoke, desert dust, sea salt) in the atmosphere. AOD is related to PM_2.5_ concentrations as measured by ground monitors, but the relationship is complex and depends on a number of other factors [3]. Popular approaches to predicting ground-level PM_2.5_ concentration using AOD include chemical-transport models, mixed-effect models, geographically weighted regression, and land-use regression, which use additional PM_2.5_ predictors and modifiers of the PM_2.5_–AOD relation such as weather and land use [3, 4]. Among the most comprehensive efforts to reconstruct ground concentrations of PM_2.5_ is NASA’s Global Modeling Initiative (GMI) chemistry transport model integrated with Modern-Era Retrospective analysis for Research and Applications, Version 2 (MERRA-2 GMI), which estimates the global distribution of PM_2.5_ mass concentrations with a spatial resolution of 0.5° × 0.625°, and temporal resolution as fine as 1 h [5, 6].

Predicting ground-based PM_2.5_ from satellite AOD retrievals is difficult. AOD is strongly influenced by particles above the surface layer, which have different characteristics from ground-level particles. Also, AOD retrieval algorithms assume consistent particle size distributions within large regions, such as Mexico and Central America [7]. Furthermore, AOD often has gaps in spatial coverage due to clouds, snow, or ice. Thus, researchers must often impute missing AOD [8], and the complex relationship between AOD and PM_2.5_, along with the use of additional PM_2.5_ predictors, has motivated machine-learning approaches such as neural networks, random forests, and gradient boosting [4, 9–12]. Given the challenges to develop a single model that fits large heterogeneous regions (e.g., national models), ensemble models combining the outputs from different machine learning algorithms have been used in recent studies [9].

AOD-based PM_2.5_ (AOD-PM_2.5_) models and predictions have allowed epidemiologists to move away from exposure-assessment methods that rely on proximity to sparse ground monitors. With sufficient spatiotemporal resolution, AOD-PM_2.5_ models may further improve exposure assessment in epidemiologic research by picking up the effects of microenvironments. Few AOD-PM_2.5_ models exist for middle-income countries. Our group developed one of the first AOD-PM_2.5_ models using daily Multi-Angle Implementation of Atmospheric Correction (MAIAC) spectral AOD derived from the Moderate Resolution Imaging Spectroradiometer (MODIS) instrument on NASA’s Aqua satellite at a 1 × 1 km spatial resolution, along with data from ground monitors, land use, and meteorological features [7]. Our previous model for the Mexico City region provides daily PM_2.5_ predictions from 2004–2014, and those predictions have been used in several epidemiologic studies in this region [13]. However, model improvements are needed to better characterize the spatiotemporal distribution of PM_2.5_, particularly since the Mexico City Metropolitan Area has undergone considerable urban sprawl. PM_2.5_ in large metropolitan areas affects not only people in the city center but also people in its suburban and rural outskirts [14]. People in the outskirts, where air-quality information is scarce, may face disproportionate health risks due to lower socioeconomic status and less access to healthcare. This environmental injustice can be even more pronounced in low- and middle-income regions [15].

AOD-PM_2.5_ models covering large urban areas have great value for epidemiology, but also for public-health surveillance (e.g., quantifying mortality and morbidity attributable to PM_2.5_) [16], environmental regulation (e.g., assessment of compliance with air quality standards) [17], and risk communication (e.g., designing air-quality indices) [18]. Furthermore, AOD-PM_2.5_ models can help air-quality administrators to see trends in the spatiotemporal distribution of PM_2.5_, map hotspots in regions with few monitors, identify emissions sources to consider for abatement actions, as well as forecast and surveillance of air pollution contingencies and wildfires [19]. Overall, AOD-PM_2.5_ models can be powerful aids for decision-making.

Most of the satellite-based PM_2.5_ models yield predictions of 24-h mean concentrations, perhaps driven by traditional approaches in epidemiology that have focused on this exposure metric, which in turn support standards for daily PM_2.5_ levels. There is growing interest in identification of specific sub-daily PM_2.5_ exposures (e.g., peak concentrations) that may trigger the onset of adverse health outcomes and harm vulnerable people. To our knowledge, this is the first model reconstructing sub-daily PM_2.5_ concentrations in Mexico.

In this study, we present a new model based on extreme gradient boosting (XGBoost) and inverse-distance weighting (IDW) that uses satellite and land-use variables to predict daily mean and max PM_2.5_ concentrations in Central Mexico. We use predictions from our models for novel and policy-relevant analyses of the determinants and distribution of population exposures.

## Methods

We constructed and evaluated two models: one for daily mean PM_2.5_, spanning 2004 through 2019, and one for daily max PM_2.5_ (i.e., the greatest hourly concentration of PM_2.5_ observed each day), spanning 2011 through 2019. We restricted our max PM_2.5_ predictions to 2011 onwards because of greater coverage of ground monitoring stations. Days were defined according to UTC − 6, which coincides with the local time of the study region (Mexico’s Zona Centro) when daylight-saving time is not in effect (namely, before the first Sunday of April and after the last Sunday of October).

### Study region

**Fig. 1:**
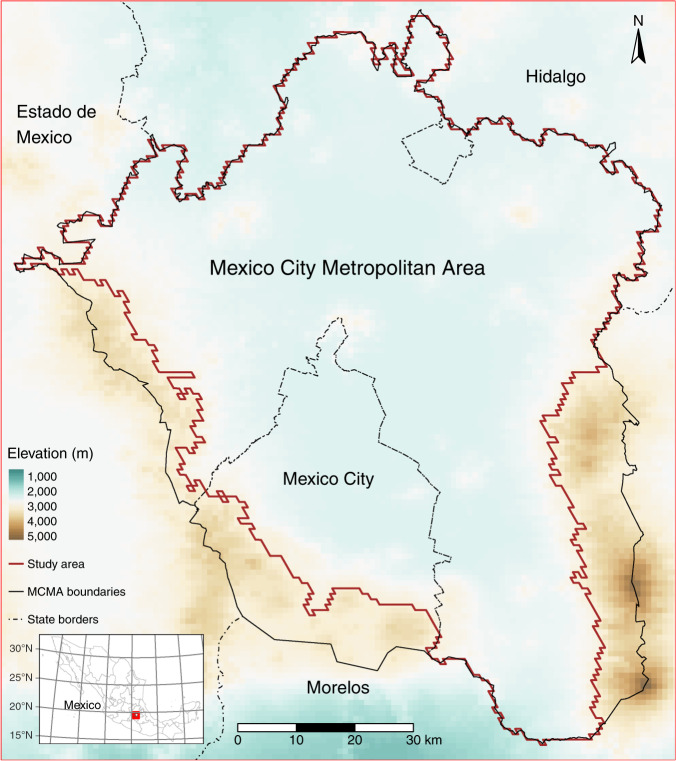
Map of study area in Central Mexico. The study area used for our PM_2.5_ models in the Mexico City Metropolitan Area (MCMA).

We modeled an irregularly shaped area of 6650 km^2^, 127 km in diameter, around Mexico City. The model used a grid of 7745 square cells, 927 m on a side, in a global sinusoidal projection (the same one used for NASA’s MODIS products). This study area and its grid was a subset of that considered in our ambient temperature model for Central Mexico [20]. We built the subset by finding the largest connected set of cells in the Valley of Mexico with all cells ≤3 km above sea level (Fig. 1). The Valley of Mexico is a plateau with a mean elevation of 2250 m above sea level, and is surrounded on three sides by mountain ranges, preventing the dispersion of air pollutants [21].

### Data

We used PM_2.5_ data from ground monitoring stations organized by the Instituto Nacional de Ecología y Cambio Climático de México (INECC) including records from the Automated Atmospheric Monitoring Network (RAMA) from the Mexico City’s Atmospheric Monitoring System (SIMAT, website http://www.aire.cdmx.gob.mx/). We downloaded observations from INECC’s website (http://scica.inecc.gob.mx). For each station in the study area and day of PM_2.5_ observations, we computed the mean and max PM_2.5_ among the hourly observations, so long as there were at least 18 h of observations in the day. Other station-days were discarded. The result was a total of 60,365 station-days from 25 stations for mean PM_2.5_ and 40,819 station-days from the same 25 stations for max PM_2.5_. The number of days of observations contributed per station ranged from 266 to 5198 (median 2030) for mean PM_2.5_, and from 50 to 2901 (median 1753) for max PM_2.5._

Our models used the following 14 predictors:

* Longitude and latitude in degrees

* The date, as an integer count of days

* The IDW mean (exponent 2) of all observations of the same dependent variable (i.e., mean PM_2.5_ or max PM_2.5_) on the given day

* MAIAC AOD from NASA’s Terra and Aqua satellites [22], with 1 km spatial resolution, whose local overpass times range from 10:40 to 15:15 and 13:10 to 15:05, respectively. We used the primary MCD19A2 product of AOD at 470 nm.

* PM_2.5_ (μg/m^3^) as predicted by MERRA-2 GMI at the surface level, with ~50 km spatial resolution [6], either the mean of the day’s 24 hourly values (for modeling mean PM_2.5_) or the value at 10:00 UTC − 6 (for max PM_2.5_)

* Temperature (K), precipitation (mm), and vapor pressure (Pa) from Daymet [23] with 1 km spatial resolution, and the temperature being computed as the mean of Daymet’s maximum and minimum temperature

* The height of the planetary boundary layer (m) and meridional and zonal wind speeds (m/s) from the 5th generation reanalysis of the global climate dataset (ERA5) of the European Center for Medium-Range Weather Forecasts (ECMWF), was downloaded from the Copernicus Climate Change Service (C3S) Climate Data Store [24], using the mean of the day’s 24 hourly values (for mean PM_2.5_) or the value at 10:00 UTC − 6 (for max PM_2.5_), with ~30 km spatial resolution

* The density of roads (m/km^2^) from OpenStreetMap [25], considering only primary, secondary, residential, and tertiary roads.

We selected the midmorning time of day 10:00 UTC − 6 in constructing some of the predictors for the max PM_2.5_ model because it was the most frequent hour of greatest daily per-station PM_2.5_ concentration in our sample.

### Model evaluation

We evaluated models with leave-one-station-out cross-validation (CV). There are 25 stations, so for each dependent variable, we fit the model 25 times, leaving out one station from training and then testing the model’s predictions on the left-out station. We evaluated models with absolute loss rather than squared loss so as not to overweight the importance of a minority of very high observed concentrations of PM_2.5_. Absolute loss leads to mean absolute error (MAE) as a natural measure of predictive accuracy (in place of root mean square error, RMSE, for squared loss), and mean absolute deviation from the median (MAD) as a measure of baseline variation in place of the standard deviation (SD) for squared loss. Note that R^2^, which is often used for model assessment, is defined as a squared-loss metric. For our study, we compute R^2^ as 1 minus the MSE divided by the variance, and we show R^2^, RMSE, and SD in tables for completeness, although the models are more properly judged in terms of absolute loss.

When computing the IDW predictor during CV, we excluded the held-out station to avoid data leakage.

### Models

We predicted PM_2.5_ with XGBoost [26], a scheme for fast boosted decision trees. We used a log-cosh objective function to approximate absolute loss. Instead of providing PM_2.5_ as the dependent variable to XGBoost directly, we provided PM_2.5_ minus the IDW interpolation and added the IDW back to XGBoost’s predictions. This method partly smooths out the otherwise discrete predictions produced by decision trees. We tuned XGBoost with twofold station-wise CV; during the larger CV discussed above, this CV was nested within each fold. Tuning adjusted four hyperparameters:

* The number of trees, which could be 10, 25, 50, or 100

* The maximum tree depth, which could be 3, 6, or 9

* The learning rate η, which could range from 0.01 to 0.5

* A ridge penalty λ, which could range from 2^−10^ to 2 [10].

We preselected a set of 25 random vectors from this space with a maximin Latin-hypercube sample using the function ‘maximinLHS‘ from the R library ‘lhs‘, version 1.1.3 [27].

Once the outer CV was done, to make new predictions, we trained the two models (one for mean PM_2.5_ and one for max PM_2.5_) on all the data, with one more tuning CV apiece. These final models had the following hyperparameters, obtained from the aforementioned tuning procedure: for mean PM_2.5_, 10 trees, max depth 3, η = 0.047, λ = 10; for max PM_2.5_, 25 trees, max depth 9, η = 0.073, λ = 260.

### Applications

We present three applications of our PM_2.5_ predictions for the Mexico City Metropolitan Area. We examined co-occurring exposures to high PM_2.5_ concentrations and high temperatures from our published spatiotemporal model [20]. Person-time estimates of exposure relied on population density estimates for 2010. We estimated the population density within each of our grid cells using the R package exactextractr [28] to calculate the area-weighted mean of the population density in the intersecting Gridded Population of the World (GPWv4) ~1-km raster cells [29]. The GPWv4 used data from the 2010 census in Mexico at the level of Área Geoestadística Básica (AGEBs; the Mexican equivalent of US Census tracts). When comparing exposures to permissible annual limits, we computed “yearly” means as the means of four 3-month means, per the Mexican standard [30]. Finally, we examined how AGEB-level PM_2.5_ exposure varied with social marginalization within the study region [31]. Every AGEB was assigned the mean PM_2.5_ prediction of all 1 × 1 km grid cells whose centroids fell within the AGEB polygon.

We used R 4.2.0 [32] with package xgboost 1.4.1.1 [33] for analysis.

## Results

Overall, the observed PM_2.5_ that we trained and tested on had a median of 23 μg/m^3^ (MAD = 8.55, IQR = 14.08) for mean PM_2.5_, and a median of 44 μg/m^3^ (MAD = 15.64, IQR = 25.00) for max PM_2.5_.

The model for mean PM_2.5_ achieved a MAE of 3.68 μg/m^3^ (compared to a MAD of 8.55 μg/m^3^), and the model for max PM_2.5_ achieved a MAE of 9.20 μg/m^3^ (compared to a MAD of 15.64 μg/m^3^). These differences indicate a substantial improvement in accuracy compared to assigning the median exposure to all places and times throughout the study domain. The much greater MAE for max PM_2.5_ than mean PM_2.5_ is to be expected, because maxima are inherently more difficult to predict than means. Tables 1 and 2 show the performance of these models stratified by year.

**Table 1:** Assessment of cross-validated predictions from the daily mean PM_2.5_ model by year.

Year	Number of stations	Observations	R^2^	SD	RMSE	MAD	MAE
2004	8	2751	0.76	12.02	5.86	9.12	3.91
2005	8	2701	0.81	14.80	6.43	11.28	4.38
2006	8	2685	0.68	13.19	7.48	9.55	5.04
2007	9	2855	0.71	10.87	5.85	8.16	4.28
2008	9	3040	0.64	12.16	7.29	9.27	4.61
2009	9	2670	0.75	10.14	5.09	7.71	3.61
2010	9	2844	0.79	11.70	5.41	8.83	3.64
2011	12	3019	0.77	11.53	5.56	8.90	3.88
2012	13	4025	0.76	10.10	4.95	7.63	3.59
2013	13	4362	0.80	11.75	5.25	8.85	3.87
2014	14	4203	0.73	9.87	5.10	7.50	3.86
2015	19	5194	0.77	10.78	5.11	7.90	3.76
2016	17	5307	0.83	11.44	4.73	8.56	3.37
2017	17	4901	0.80	10.79	4.78	8.42	3.15
2018	17	4633	0.84	9.91	3.94	7.19	2.83
2019	20	5175	0.86	11.50	4.26	7.98	2.85

*SD* Standard deviation, *RMSE* Root mean squared error, *MAD* Mean absolute deviation, *MAE* Mean Absolute Error.

**Table 2:** Assessment of cross-validated predictions from the daily 1-h maximum PM_2.5_ model by year.

Year	Number of stations	Observations	R^2^	SD	RMSE	MAD	MAE
2011	12	3019	0.47	24.26	17.63	16.65	10.36
2012	13	4025	0.46	21.80	16.09	15.18	10.17
2013	13	4362	0.58	23.78	15.49	17.28	10.27
2014	14	4203	0.52	19.54	13.58	14.46	9.76
2015	19	5194	0.63	25.30	15.34	16.37	9.97
2016	17	5307	0.62	25.33	15.59	16.48	8.68
2017	17	4901	0.56	23.86	15.75	15.85	8.48
2018	17	4633	0.63	19.68	11.96	13.74	7.83
2019	20	5175	0.66	21.39	12.50	14.08	8.04

*SD* Standard deviation, *RMSE* Root mean squared error, *MAD* Mean absolute deviation, *MAE* Mean Absolute Error.

We also compared model performance by season: cold dry (spanning November through February), warm dry (March to May), and rainy (June to October) [34]. Supplementary Table 1 shows that the largest improvement in prediction accuracy (MAD minus MAE) was observed during the cold dry season for both mean and max PM_2.5_ models, although this season still had the largest MAE.

Supplementary Table 2 shows the Pearson correlations among observed and predicted PM_2.5_ for both models. As would be expected, all four variables are positively related. Predictions are more associated with the kind of observation they are meant to predict than the other kind, but there are also strong correlations between mean and max PM_2.5_.

**Fig. 2:**
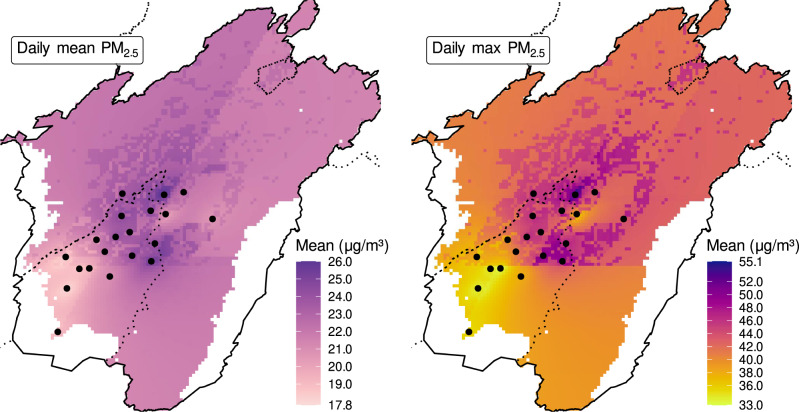
Maps of the averaged annual daily mean and daily max PM_2.5_ concentrations for 2019 in the Mexico City Metropolitan Area. Solid and dotted lines indicate the Mexico City Metropolitan Area and Mexican states boundaries, respectively. Black dots indicate ground monitors.

After making predictions for every grid cell and day with both models, we mapped the per-cell mean PM_2.5_ and max PM_2.5_ averaged over 2019 (Fig. 2). Discontinuities in the prediction surfaces evident in our maps are the result of model-based splits selected in the longitude and latitude predictors. Although we also include an IDW interpolation that adds some smoothness, XGBoost selects for the most predictively accurate model. Smoothing our predictions more aggressively could make for more intuitive maps, but would not necessarily improve predictive accuracy. As expected, the highest concentrations (shown in dark purple) are in the center-north and center-east subregions of the Mexico City Metropolitan Area (north and east of Mexico City, respectively), with the highest population density and industrial land use. This pattern is also visible in the max PM_2.5_ map, but is most pronounced in the center-north. The lowest PM_2.5_ concentrations (shown in light purple and yellow) are in the southwest, corresponding to the least populated and the most vegetated subregion.

We examined the per-day ratio (collapsing across all cells) of mean and max PM_2.5_. Supplementary Fig. 1 shows this ratio for each day in 2019. Generally, the max is about twice the mean, but the ratio decreases in the first half of the year and increases in the second. During the rainy season (June to October), we examined how the ratio differed between days with and without a mean per-cell precipitation of at least 1 mm, and found little difference: the mean ratio was 2.03 on dry days and 2.13 on rainy days.

**Fig. 3:**
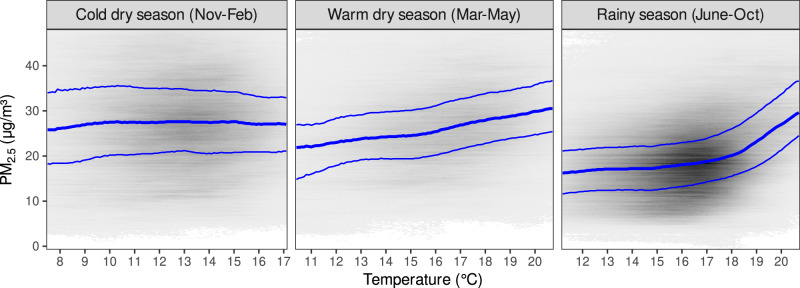
Heatmaps of mean temperature and mean PM_2.5_, counting all grid cells and days equally. Darker areas indicate more grid cells, more days, or both. Temperature and PM_2.5_ predictions are already rounded to the nearest tenth, so no further grouping is needed for a heatmap. For legibility, the temperature scale only shows the middle 95% of the data for each season, and the PM_2.5_ scale only goes up to the 98^th^ percentile for all seasons. Blue lines show the quartiles of PM_2.5_ conditional on temperature.

With our temperature model [20], we examined the relationship between mean daily PM_2.5_ and mean daily temperature. The Kendall correlation between the two over the whole study period was 0.05, indicating a very weak positive relationship overall. Figure 3 breaks this relationship down by season. It can be observed that the PM_2.5_ concentrations are more stable and remain high during the cold dry season, which has been related to the stable atmospheric conditions and frequent thermal inversions in the study region. For the warm dry season and rainy season, there is a clearer tendency for higher PM_2.5_ concentrations on hotter days.

Considering the 88,399 cell-days in which mean PM_2.5_ exceeded Mexico’s permissible daily limit of 41 μg/m^3^ [30], the median temperature was 19.2 °C, somewhat warmer than the median in all other cell-days, 15.9 °C. Considering the 173,170 cell-days with a mean temperature of at least 20 °C, we found substantially higher median PM_2.5_, 30.2 μg/m^3^, than in all other cell-days, 19.7 μg/m ^3^.

**Fig. 4:**
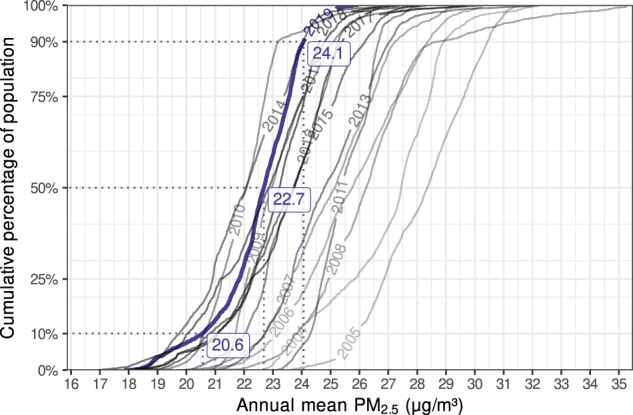
Population estimated annual average exposures. The figure shows an empirical cumulative distribution curve for each year from 2004 to 2019, generated from our daily mean model and using the 2010 census population density. Specific quantiles are labeled for the year 2019, where only 10% of the population in the study region had an annual average exposure below 20.6 μg/m³.

We used population density from GPWv4 in every prediction cell of the study area to estimate person-days of PM_2.5_ exposure in 2010, referring to Mexico’s standards for annual and daily ambient concentrations of PM_2.5_ [30]. We compared the exposure estimated by our XGBoost-with-IDW model to that estimated by IDW alone, a PM_2.5_ interpolation technique that has been used for a health-impact assessment in this region [35]. The study area contained 20,279,491 people in 2010. According to both our model and the IDW-only model, every single person in the Mexico City Metropolitan Area experienced a yearly mean PM_2.5_ worse than the permissible limit of 10 μg/m^3^. The large majority of people (97%, or more than 99% according to IDW) experienced a yearly mean more than twice the limit. Similarly, all people experienced at least one day with a mean PM_2.5_ worse than the daily permissible limit of 41 μg/m^3^. People experienced a mean of 21.6 (23.7 according to IDW) days exceeding the limit. The total number of exceeded person-days was 439 million (481 million according to IDW). Overall, we find widespread exposure to worse-than-permissible air pollution, although our full model suggests slightly less exposure than an IDW-only model. To show population exposure distributions over time, we also calculated the annual average concentration for each populated grid cell for each year, using more than 45 million model predictions. Figure 4 shows the empirical cumulative distribution functions for these annual concentrations calculated with 2010 census population densities. As observed in Fig. 4, there has been an overall reduction in the annual exposure to PM_2.5_ since the earliest years (2004 and 2005); however, there is considerable variability in the estimated annual exposures, with less clear recent trends.

We used an index of social marginalization developed by the Consejo Nacional de Población (CONAPO), which considers access to education and health, housing characteristics, and possession of goods [31], to compare urban marginalization in 2010 to mean PM_2.5_. There were 2,065 AGEBs with available marginalization scores (AGEBs’ median area was 0.46 km^2^, range 0.014 to 7.4 km^2^), with one score per AGEB and year, so we summarized mean PM_2.5_ in 2010 by AGEB. Overall, marginalization and PM_2.5_ were Kendall-correlated 0.024, which is a relationship in the expected direction (i.e., AGEBs with more marginalized populations being exposed to more air pollution), but very weak. Breaking the AGEBs into 0.5-unit groups of marginalization (with one group for marginalization −2 to −1.5, one for −1.5 to −1, etc.), we find a small range of mean per-group PM_2.5_, from 21.78 to 22.56 μg/m^3^.

## Discussion

We constructed and validated models to predict mean and max PM_2.5_ in the Mexico City Metropolitan Area, and examined potential applications in air-pollution epidemiology and air-quality management. Our machine-learning-based model is the first of its kind in Mexico, although previously, our team used mixed-effects models with AOD to predict mean PM_2.5_ in this region [13]. Also new is our consideration of max PM_2.5_, an exposure metric that is becoming relevant to address subdaily health effects from peak exposures to PM_2.5_ [36]. Overall, our models exhibited good performance, with prediction errors that decreased over time, as the number of ground monitoring stations increased. Our per-year R^2^ for mean PM_2.5_ ranged from 0.64 to 0.86, similar to the R^2^ values for our team’s XGBoost model in the Northeastern US, which ranged from 0.64 to 0.80 [11]. Our new modeling approach could be extended to other regions with low or intermediate density of ground monitoring stations.

Recently the ensemble model framework has become a popular approach to combine PM_2.5_ estimates from different machine-learning models, mostly in data-rich regions where ensemble models have utilized tens to over 100 predictors [9]. The implementation of ensemble models in sparsely monitored regions like the Mexico City Metropolitan Area would be challenging because it typically requires withholding more data in order to construct model weights. Despite their potential benefits, the incremental performance from ensemble models compared to single machine-learning algorithms has been reported as small, especially when the base learners perform well (e.g., R^2^ > 0.7), and the same predictors are involved [9]. Overall, the performance of our XGBoost model to predict mean PM_2.5_ was good, and similar to the performance of other tree-based models using a single learner [10], or ensembles using XGBoost [37] or not [9] as one of their learners. Boosted trees (fitting trees sequentially to the residual error of the prior ensemble) typically outperform the independent trees in random forests. XGBoost’s multiple forms of regularization help to avoid overfitting and achieve high accuracy and it is often a best-in-class predictive algorithm with smaller datasets [26, 38].

PM_2.5_ predictions from AOD-PM_2.5_ models have been used in epidemiology to reduce exposure measurement error, but may also be useful for applications such as air-quality management, particularly in sparsely monitored regions [19]. Figure 2 shows wide variation in both PM_2.5_ metrics across the Mexico City Metropolitan Area. More PM_2.5_ has historically been observed in the center-north and center-east (in the densely populated limits between Mexico City and the State of Mexico), where there are substantial emissions from industry and traffic [39]. Our PM_2.5_ predictions allowed us to assess exposure to PM_2.5_ in the entire Mexico City Metropolitan Area, unlike previous studies that could only partly cover this region with data from ground monitoring stations alone [35]. The estimated annual mean concentrations from our model exceeded the current annual PM_2.5_ Mexican permissible limits across the entire study region, supporting previous results pointing out that despite significant improvements in the air quality of Mexico City for PM_10_ and ozone since the 1990s, there remain substantial obstacles to reducing emissions of PM_2.5_ and its precursors [40]. The use of our spatiotemporally resolved PM_2.5_ predictions should improve future health impact assessments and support targeted exposure reduction strategies in this region [41].

Seasonally, there is a well-defined pattern of higher PM_2.5_ concentrations during the two dry seasons (Nov-May), due to frequent thermal inversions and stable atmospheric conditions, which favors the accumulation of PM_2.5_. The lowest PM_2.5_ concentrations occur during the rainy season (June-Oct), due to wet deposition [42]. We hypothesized that the observed pattern in the daily ratios of mean and max PM_2.5_ (Supplementary Fig. 1) reflects the influence of seasonal meteorological conditions. We checked whether higher ratios observed during the rainy season could be explained by precipitation, since late-afternoon showers can reduce PM_2.5_ [42]. However, we found that days with at least 1 mm of daily precipitation had only a 5% greater ratio than other days. Evidence from cities at high elevations (>2000 m above sea level) has shown that relative humidity interacts with precipitation and PM_2.5_ emission sources to increase or decrease PM_2.5_ concentrations [43]. Increasing relative humidity can raise PM_2.5_ concentration depending on the PM_2.5_ composition and hygroscopic growth ability, especially in traffic-heavy residential areas where only strong rain events (e.g., precipitation >9 mm) are effective in removing PM_2.5_ from the atmosphere. In industrial areas, high relative humidity conditions are more important to decrease PM_2.5_ concentrations, regardless of rain events. Weak rain episodes (e.g., precipitation <1 mm), can also increase PM_2.5_ concentrations by worsening traffic in rush hours and reducing combustion efficiency [43]. It is possible that the ratios of mean and max PM_2.5_ observed in Supplementary Fig. 1 are produced by the interaction of precipitation, humidity, and PM_2.5_ sources in the study region.

In the context of climate change, it is important to characterize the increasingly common joint occurrence of extreme air pollution and extreme temperatures [44]. We found that while PM_2.5_ and temperature are only weakly related overall, higher PM_2.5_ concentrations tended to occur on warmer days, particularly in the rainy season (Fig. 3), and conversely, days with mean temperatures of at least 20 °C had a substantially worse median PM_2.5_ concentration than cooler days. It has been reported that co-occurring extreme PM_2.5_ and extreme temperatures may increase the acute risk of illness [45], and that the influence of PM_2.5_ on mortality rates may be stronger in warmer cities [46]. Previous studies in the Mexico City Metropolitan Area have suggested stronger associations with mortality on days with high PM_2.5_ and extreme temperatures [47], but they may have estimated effects imprecisely, given their citywide approach for estimating exposure. Our PM_2.5_ predictions can improve exposure assessment and air-pollution epidemiology, including studies addressing the interactive effects of PM_2.5_ with temperature.

To put into perspective the human cost of PM_2.5_ exposure, we found that in 2010, every person in the study region was exposed to unhealthy air quality according to the Mexican standards for annual (10 μg/m^3^) and daily (41 μg/m^3^) concentrations, which are several times the recently enacted World Health Organization Guidelines of 5 and 15 μg/m^3^, respectively [48]. Overall, in 2010 the population of the study region experienced a mean of nearly 3 weeks of PM_2.5_ above the current daily Mexican permissible limit. For epidemiologic research, the distribution of continuous exposures is more relevant for health studies than the dichotomous assessment or duration of compliance with a particular standard. The annual empirical cumulative distributions for all inhabited areas in the study region in Fig. 4 are a summary of the population distribution of our exposure estimates that is suitable for assessment of long-term ambient PM_2.5_ exposures and related chronic health effects.

Concentrations of PM_2.5_ measured in a single monitoring station are used to represent the pollution conditions over large spatial domains (up to tens of kilometers) for a specific amount of time, such as one day or one year. However, PM_2.5_ levels can be rapidly influenced by local sources, increasing not only concentrations between monitoring sites, but also the risks of acute health effects. A distinguishing feature of our model is that we also generated a sub-daily metric of PM_2.5_ concentrations, namely, max PM_2.5_ at a 1-km resolution. There are not yet any air-quality standards for sub-daily PM_2.5_ concentrations, but new research into the health impacts from such exposures could eventually support new standards [49, 50]. The US Environmental Protection Agency states that *“*Because a focus on annual average and 24-h average PM_2.5_ concentrations could mask sub-daily patterns, and because some health studies examine PM exposure durations shorter than 24-h, it is useful to understand the broader distribution of sub-daily PM_2.5_ concentrations” [36]. Because it’s more difficult to reconstruct extrema (e.g., max PM_2.5_) than measures of central tendency (e.g., mean PM_2.5_), future work on estimating health impacts from max PM_2.5_ could particularly benefit from estimating and propagating prediction uncertainty into downstream analyses [51].

Our comparison of PM_2.5_ exposure across levels of social marginalization did not suggest meaningful differences between groups. However, the 2010 Mexican index of social marginalization was only available for urban AGEBs: those with a total population of more than 2500. Without data for rural AGEBs or irregular settlements, it is naturally more difficult to assess the influence of socioeconomic status. Since the methods employed in the construction of the Mexican index of social marginalization have changed over time, it would be difficult to analyze multiple years and make sense of the differences between them. One study found that in Mexico City in 2015, per-AGEB deprivation was positively associated with PM_10_, but negatively associated with ozone [14]. In this region, PM_10_ concentrations are highly influenced by local emissions from point and area sources (mainly unpaved roads), which may explain why PM_10_ was associated with deprivation. However, PM_2.5_ is strongly influenced by mobile sources, and most of the PM_2.5_ concentrations are secondary aerosols that can travel far from their emission sources, leading to homogeneous PM_2.5_ concentrations [39]. AGEBs are the smallest geographic units with information on marginalization scores, and homogeneous socioeconomic characteristics are expected within AGEBs. Nonetheless, it is also possible that socioeconomic variation exists within AGEBs given the large variability in their size, which might affect correct classification of unequally exposed groups.

Despite the good performance of our models throughout the study period, we observed seasonal differences in their performance, which have also been reported in other studies [9, 10, 12]. This suggests that seasonal differences are less a property of our model than a property of the data. The implications of these seasonal differences on the accuracy of PM_2.5_ predictions for exposure assessment in epidemiologic research should be addressed in future studies. Also, as in any other PM_2.5_ prediction strategy, our models depend on the location of ground monitors, which may be not representative of the entire study area; therefore, error in PM_2.5_ prediction can arise especially in remote locations.

A particular limitation of our max PM_2.5_ model arises from the limited temporal resolution in the AOD data. Each satellite passes over the Central Mexico region only once during each period of daylight, possibly missing sudden episodes of intense PM_2.5_. However, the overpass time of the Terra satellite is similar to the daily peak of PM_2.5_ according to ground monitoring stations, so in general, Terra AOD should be representative of max PM_2.5_. Future work will utilize AOD data from the Advanced Baseline Imager (ABI) aboard NOAA’s Geostationary Operational Environmental Satellite-R Series (GOES-16 and GOES-17) with temporal resolution as high as 5 min over Mexico City. Synergistic AOD products developed from the ABI and upcoming NASA geostationary Tropospheric Emissions: Monitoring of Pollution (TEMPO) mission, planned for launch in 2023, will further enhance capabilities to predict and monitor PM_2.5_ concentrations in the region. TEMPO will advance exposure science in North America, particularly by providing hourly observations of aerosols and gaseous pollutants for supporting air-pollution models [52, 53].

## Supplementary information

Reporting Checklist

Supplemental Material

## Data Availability

The authors confirm that the data supporting the findings of this study are thoroughly described in the article and links are provided in the reference section. Derived data supporting the findings of this study are available from the corresponding author on request.
