# Supplementary material for: Prediction of daily mean and one-hour maximum PM2.5 concentrations and applications in Central Mexico using satellite-based machine-learning models
Source: J Expo Sci Environ Epidemiol. 2022 Sep 10;32(6):917–25. doi: 10.1038/s41370-022-00471-4 (PMC9731899; doi:10.1038/s41370-022-00471-4)
Supplement: Supplementary file 2 — Supplemental Material [file 41370_2022_471_MOESM2_ESM.docx]

**Supplemental Material**

**Title:** Prediction of daily mean and one-hour maximum PM_2.5_ concentrations and applications in Central Mexico using satellite-based machine-learning models

Iván Gutiérrez-Avila*^1^, Kodi B. Arfer^1^, Daniel Carrión^1,2,3^, Johnathan Rush^1^, Itai Kloog^1,4^, Aaron R. Naeger^5^, Michel Grutter^6^, Víctor Hugo Páramo-Figueroa^7^, Horacio Riojas-Rodríguez^8^, Allan C. Just^1,9^

**Author Affiliations:**

^1^Department of Environmental Medicine and Public Health, Icahn School of Medicine at Mount Sinai, New York, NY, USA

^2^Department of Environmental Health Sciences, Yale University School of Public Health, New Haven, CT, USA

^3^Center on Climate Change and Health, Yale University School of Public Health, New Haven, CT, USA

^4^Department of Geography and Environmental Development, Ben-Gurion University of the Negev, Beer Sheva, Israel

^5^Earth System Science Center, University of Alabama in Huntsville, Huntsville, AL, USA

^6^Instituto de Ciencias de la Atmósfera y Cambio Climático, Universidad Nacional Autónoma de México, Ciudad de México, México

^7^Comisión Ambiental de la Megalópolis, Ciudad de México, México

^8^Dirección de Salud Ambiental, Instituto Nacional de Salud Pública, Cuernavaca Morelos, México

^9^Institute for Exposomic Research, Icahn School of Medicine at Mount Sinai, New York, NY, USA.

**Number of tables:** 2

**Number of figures:** 1

Supplementary Table 1. Assessment of cross-validated predictions for the mean and max PM_2.5_  models by season from 2004-2019

Supplementary Table 2. Correlation coefficients between observed and predicted mean and max PM_2.5_ concentrations

Supplementary Figure 1

Supplementary Table 1. Assessment of cross-validated predictions for the mean and max PM_2.5_

models by season from 2004-2019


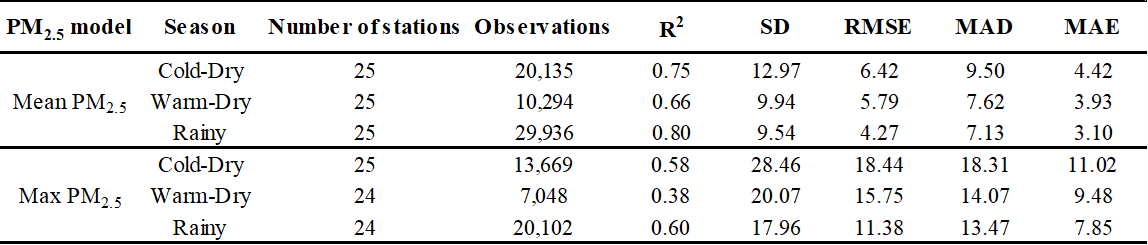


Standard deviation (SD), Root mean squared error (RMSE), Mean absolute deviation (MAD), and Mean Absolute Error (MAE). Cold-Dry (Nov-Feb), Warm-Dry (Mar-May), and Rainy (June-Oct).

Supplementary Table 2. Correlation coefficients between observed and predicted

mean and max PM_2.5_ concentrations


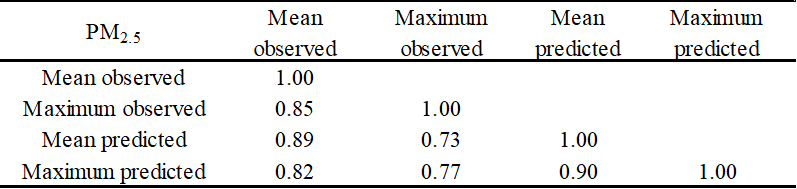


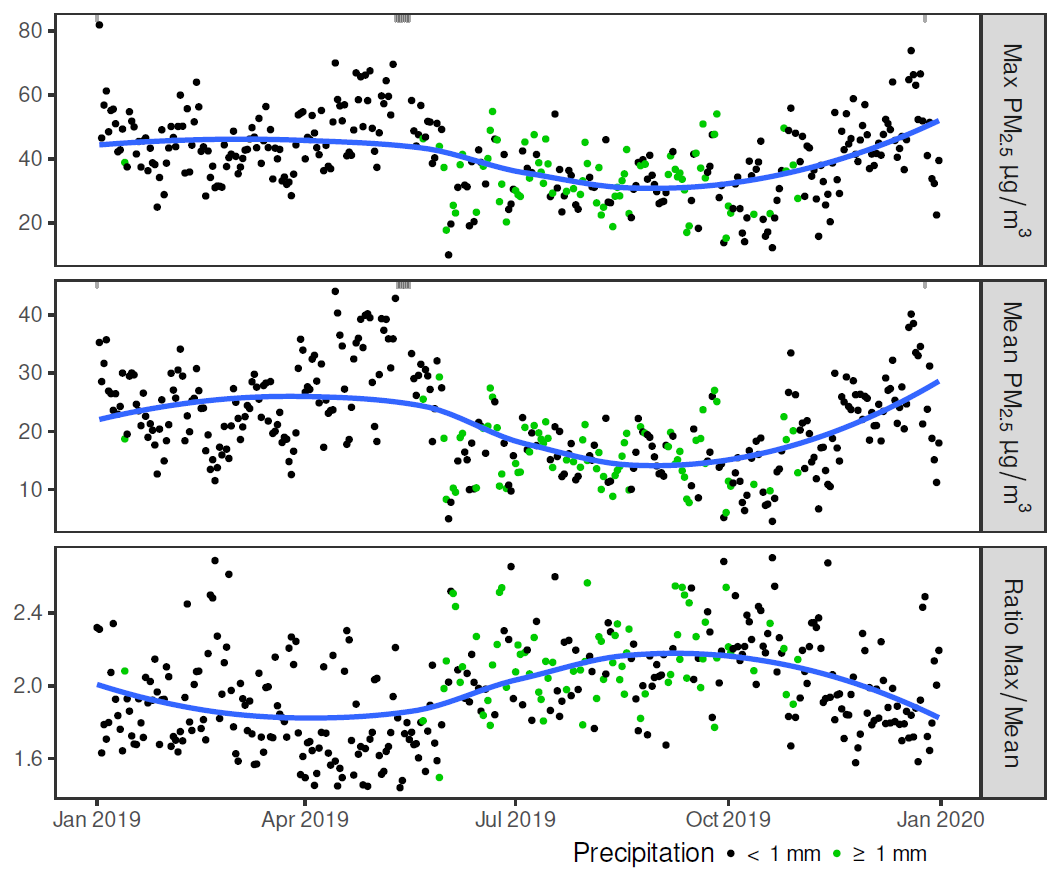


Supplementary Figure 1. For each day in 2019, the mean of three predicted quantities (max PM_2.5_, mean PM_2.5_, and max divided by mean) across all cells. A locally estimated scatterplot smoothing (LOESS) trendline is shown for each panel. Days are colored according to the mean precipitation across all cells. For legibility, nine especially high points (> 90 μg/m^3^) are excluded in the max panel and eight especially high points (> 45 μg/m^3^) are excluded in the mean panel (indicated by ticks on the top borders); the corresponding ratios are still included in the bottom panel.
